# Structural characterization and antioxidant activity of processed polysaccharides PCP-F1 from *Polygonatum cyrtonema* Hua.

**DOI:** 10.3389/fnut.2023.1272977

**Published:** 2023-09-05

**Authors:** Yuanyuan Zhao, Zhen Wang, Ranze Fu, Ruonan Xie, Bin Wang, Qinglin Li

**Affiliations:** ^1^Key Laboratory of Xin'an Medicine of the Ministry of Education, Anhui University of Chinese Medicine, Hefei, China; ^2^Institute of Pharmaceutical Chemistry, Anhui Academy of Chinese Medicine, Hefei, Anhui, China

**Keywords:** polysaccharide, *P. cyrtonema* Hua., steaming and shining, structural characterization, antioxidant activity, functional food

## Abstract

**Introduction:**

*Polygonatum cyrtonema* Hua. (PC) is a traditional Chinese herb with a history of use in both food and medicine. For clinical use, processed PC pieces are most commonly used, while present research has focused on crude PC polysaccharides (PCPs).

**Methods:**

In this study, a new polysaccharide, PCP-F1, with a molecular weight of 37.46 kDa, was separated from four-time processed PCPs by column chromatography and evaluated by antioxidant activity. It was composed of glucose, mannose, galactose, rhamnose, and galacturonic acid with a molar ratio of 3.5: 2.5: 1.3: 1.8: 0.8.

**Results and Discussion:**

The methylation analysis and two-dimensional NMR measurement revealed that the configuration of PCP-F1 contained nine residues in the primary structural unit by the chain of →3)-*α*-D-Glc*p*, →2)-*α*-D-Glc*p* (6→, →1)-*ꞵ*-D-Glc*p* (2→, →2)-*α*-D-GalA*p* (3,4→, →1) -*ꞵ*-D-Man*p* (3→, →2)-*α*-D-Glc*p* (3→, branched for →3)-*α*-D-Glc*p*, →2)-*ꞵ*-D-Gal*p* (4→, →1)-*ꞵ*-D-Glc*p* (2→, →2,4)-*α*-D-Man*p* (6→, →3)-*α*-L-Rha*p* (4→. Radical scavenging assays indicated that PCP-F1 could scavenge radicals with a high scavenging rate, suggesting PCP-F1 possesses good antioxidant activity. The study confirms the importance of processed PC and offers the potential for exploiting it as a functional food.

## 1. Introduction

The polygonata rhizoma “Huangjing” was first described in the Mingyi-Bielu. Among the three types of Huangjing, the rhizome of *P. cyrtonema* Hua. is the most commonly used for medicine and food (1, 2). The chemical components of *P. cyrtonema* Hua. mainly include polysaccharides, steroidal saponins, alkaloids, and so on. Notably, the significant important polysaccharides component has various bioactivity, such as enhancing human immunity (3), antitumor (4), and improving superoxide dismutase activity (5), and are usually designed as the quality marker (Q-Marker) of *P. cyrtonema* Hua. It. Although *P. cyrtonema* Hua. is a type of traditional Chinese medicine (TCM), it can only be used as medicine after steaming to ensure its safety and efficacy (6). Additionally, the steaming process eliminates some side effects such as a numb tongue, and stimulation throat; more importantly, it enhances the pharmacological function.

Currently, various processing methods for *P. cyrtonema* Hua. (PC) include single-steaming, wine-steamed, nine-steaming, and nine-basking (7, 8). These methods impact the polysaccharide compositions. “Nine-steaming and nine-basking” is a traditional approach derived from Lei-Gong-Paozhilun, later refined in Qianjin-Yi-Fang. Chemical changes occur after nine cycles, enhancing the tonifying effect and reducing throat irritation (9). Initial PC rhizomes lack GalA but gain it during steaming, with shorter steaming (2–4 h) yielding more immunologically active PCPs (10). HPLC-QTOF-MS/MS revealed an oligosaccharide in steamed PC with fructose units linked by *ꞵ*-(2 → 1) or *ꞵ*-(2 → 6) patterns (11). Steaming elevates Gal while reducing Glc and Man, and GC–MS shows increased *β*-1, 4-Man*p*, and *β*-1, 4-Gal*p*, resulting in heightened antioxidant activity (12). However, the structure–activity relationship of PCPs from the nine-steaming and nine-basking methods remains unexplored.

Reactive oxygen species (ROS) are present in living organisms and help maintain cell homeostasis (13). These compounds arise from the normal metabolism and oxidation of xenobiotics. Organisms use an antioxidant system to balance oxidative stress and protection. ROS, particularly radicals, pose a cell-damaging risk (14). To counter radical damage, organisms generate endogenous or exogenous antioxidant species. Some synthetic antioxidants like octyl gallate and propyl gallate are approved but face safety concerns (15). Notably, natural antioxidants are preferred, with hydroxyl groups of polysaccharides displaying radical scavenging and antioxidative functions (16). Moreover, properties of polysaccharides, like water solubility, size, monosaccharide composition, and structure, influence their activity (17). Given the intricate structural nature of polysaccharides in traditional Chinese medicine (TCM), marked by diverse monosaccharide types and compositions, linking modes, and molecular sizes, the task of conducting structural analyses on these polysaccharides becomes both captivating and daunting.

Following up on the above reports and continuing our interest in studying TCM (18–21), particularly *Polygonatum Rhizoma* (22–25), we reported a novel polysaccharide, PCP-F1, prepared by using the nine-steaming and nine-basking approach. By methylation analysis combined with Congo red assay, a detailed structure was determined. Furthermore, two radical scavenging models were employed to assess the antioxidant effect of PCP-F1. Lastly, the correlation between antioxidant activity and corresponding structural characteristics of PCP-F1 was revealed.

## 2. Results

### 2.1. Physicochemical properties of processed PCPs total sugar, acid sugar, and FT-IR spectroscopy

**Figure 1:**
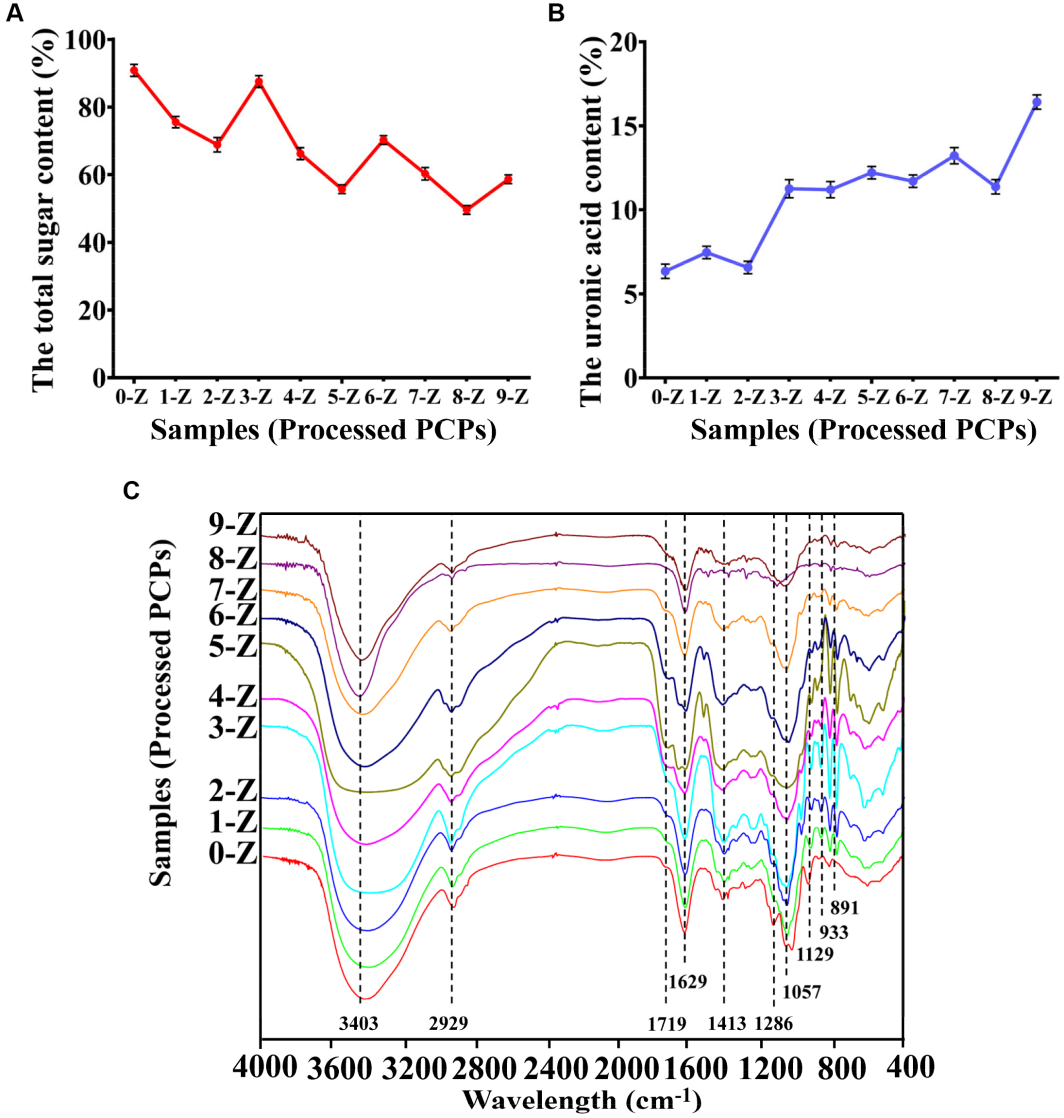
The physicochemical property analysis. The total sugar content **(A)**, the uronic acid content **(B)**, and FT-IR spectroscopy **(C)** of *Polygonatum cyrtonema* Hua. are processed by different times of repeated steaming (0-Z indicates no steam process; 1-Z–9-Z is the number of steaming times. Tested for three times at 25°C, *p* < 0.05.

The content of total sugar and acid sugar is often used as the quality control index of traditional Chinese medicine. As shown in Figure 1A, the 0-Z PCPs had the highest total sugar content of 90.87 ± 1.77%. As the number of steaming increases, the total sugar content decreases gradually. In the 2-Z PCPs, the total sugar content is 68.89 ± 2.14% and tends to stabilize at 66.24 ± 1.77% in the 4-Z PCPs. On the other hand, 0-Z PCPs had a content of acid sugar of 6.35 ± 0.43% which is the lowest in the processed PCPs, as shown in Figure 1B. In the 2-Z PCPs and the 4-Z PCPs, the content of acid sugar is 6.57 ± 0.37 and 11.20 ± 0.48%, respectively. Subsequently, increasing the number of steaming times did not cause a significant increase in the content.

The FT-IR method is commonly used for analyzing polysaccharide structures (26). By analyzing the FT-IR spectrum of processed PCPs, we were able to characterize their structural characteristics. According to Figure 1C, the spectra of processed PCPs had similar absorption characteristics, indicating that the steaming process did not significantly affect the substituent groups. It was confirmed that a wide peak of polysaccharides was located at 3,403 cm^−1^, which was attributed to the –OH group in the polysaccharides. In addition, the peak width gradually widened at 2-Z with the increase of steaming times but became sharp again from the beginning of 4-Z. The peak 2,929 cm^−1^ was assigned to the C-H asymmetric tensile vibration (27), and the weak peak located at 1,719 cm^−1^ was attributable to the vibration of symmetry C=O (28). It is noteworthy that the C-O-C glycosidic bond, as well as the C-C and C-OH stretch vibration bonds, are primarily observed between 1,200 and 1,000 cm^−1^. These two signals at 1,057 and 1,129 cm^−1^ were identified as stretching vibrations of the pyranose ring (29). In addition, the peaks at 933 and 891 cm^−1^ were ascribed to *β*-type glycosidic and *α*-glycosidic bonds, respectively (30).

### 2.2. pH measurement and antioxidant activity

**Figure 2:**
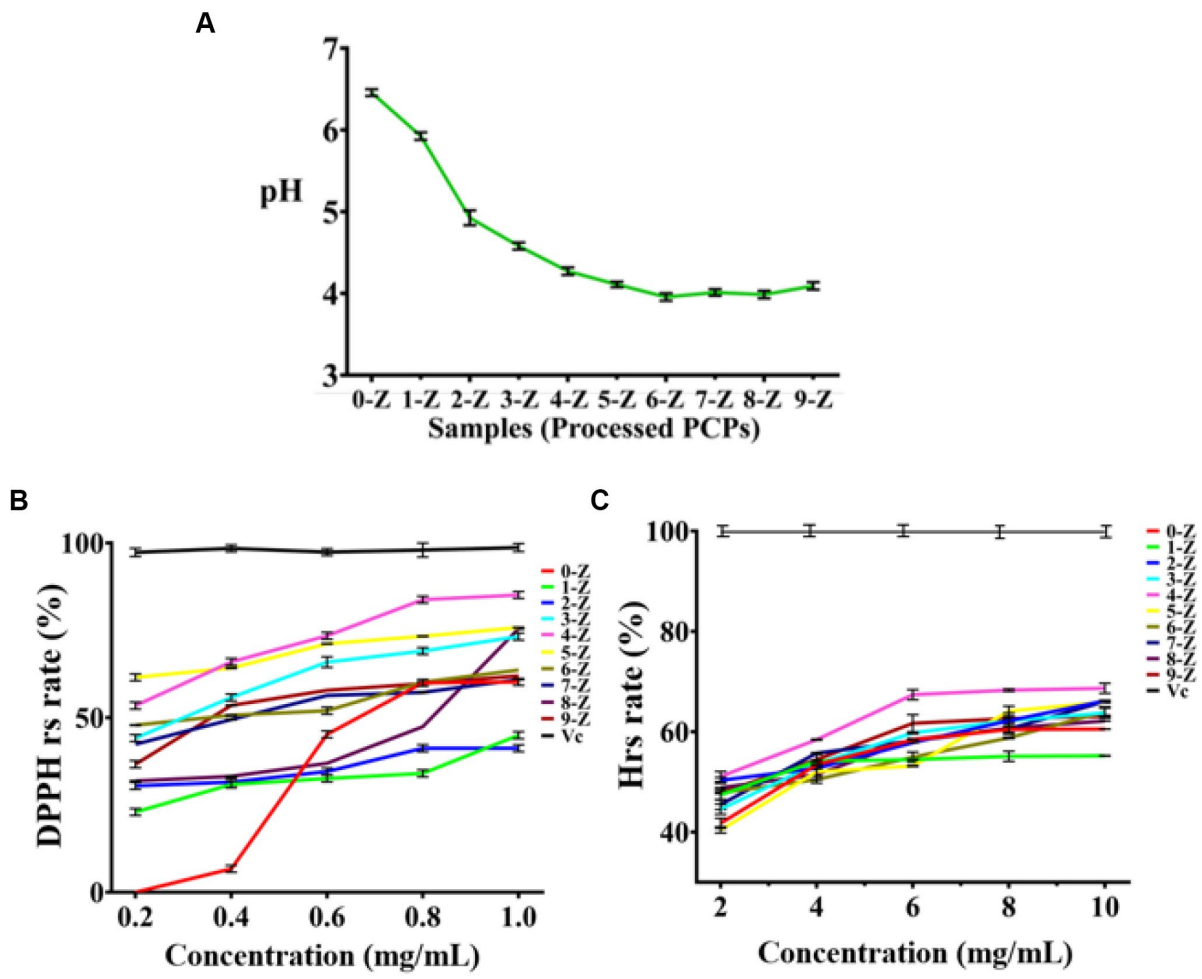
The characterization and antioxidant activities analysis. The pH analysis of different processed PCPs **(A)**, DPPH radical scavenging experiments of different processed PCPs **(B)**, and Hydroxyl radical scavenging experiments of different processed PCPs **(C)**, (Tested for three times at 25°C, *p* < 0.05).

When the pH decreased, electron transfer occurs very quickly in an ionized solution due to electron availability (31, 32). As presented in Figure 2A, the pH value of 0-Z PCPs solution was 6.46 ± 0.04, while the pH value of 3-Z PCPs solution was 4.58 ± 0.04, indicating a marked increase in the acidity of the solution. Nevertheless, the pH value was 4.27 ± 0.05 in the solution of 4-Z PCPs. Even if the number of steaming is further increased, the pH value of the corresponding solution of processed PCPs tends to stabilize around 4.0, which may be because the acid substance was gradually stable after 4-Z (33).

The occurrence of some diseases is closely related to the existence of inflammation. However, high concentrations of radicals in the body can lead to the occurrence and development of inflammation (34). Herein, we carefully investigated the antioxidant activity of 10 kinds of processed PCPs (0-Z, 1-Z, 2-Z, 3-Z, 4-Z, 5-Z, 6-Z, 7-Z, 8-Z, and 9-Z PCPs) in the range of 0.2–1.0 and 2.0–10.0 mg/mL to determine the higher activity portion. Antioxidant assays were performed by two models: scavenging DPPH radicals and hydroxyl radicals. The 10 kinds of processed PCPs showed effective antioxidant activity and the 4-Z PCPs exhibited higher radical-scavenging abilities in both models. The DPPH radical scavenging rate of processed PCPs was presented in Figures 2B,C as follows: As a positive control, the Vc group had the highest radical scavenging rate.

The scavenging rate was 0.0% ± 0.0 in the 0-Z PCPs group with the concentration was 0.2 mg/mL; increasing the concentration to 0.6 mg/mL drastically increased the scavenging rate of 0-Z PCPs to 45.2% ± 1.0, while when the concentration is 1.0 mg/mL, the scavenging result was somewhat affected. Meanwhile, 4-Z PCPs have a scavenging rate of 54.1% ± 1.4 at the concentration of 0.2 mg/mL, obviously higher than 0-Z PCPs and slightly lower than the 5-Z PCPs group. When the concentration of each processed PCPs increases to 0.6 mg/mL, the highest scavenging rate was found in the 4-Z PCPs group, and a similar result was also found when the concentration of analyte is 1.0 mg/mL. Subsequently, in the hydroxyl radical assays, we observed a similar phenomenon on the radical scavenging effect. Combining the results of the two models, 4-Z PCPs had a more robust antioxidant performance in all the processed PCPs groups. The EC50 of DPPH and hydroxyl radicals scavenging rate of 4-Z PCPs were 0.049 and 1.95 mg/mL, respectively.

### 2.3. Physicochemical properties of PCP-F1

#### 2.3.1. Antioxidant activity

**Figure 3:**
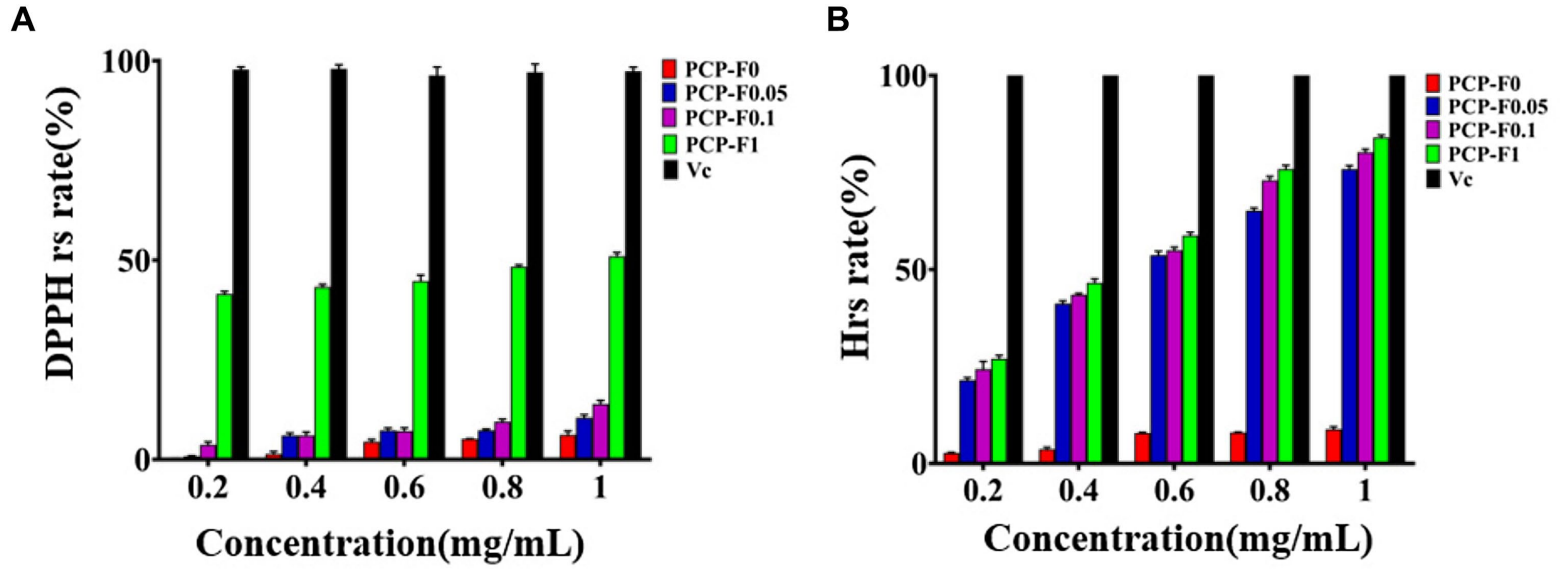
The evaluation of antioxidant activities. DPPH radical scavenging experiments **(A)** and Hydroxyl radical scavenging experiments **(B)** of PCP-F0, PCP-F0.05, PCP-F0.1, and PCP-F1, Vc as the control sample. (Tested three times at 25°C, *p* < 0.05).

Crude 4-Z PCP (64.88 g) was carefully isolated and then purified from the rhizomes of *P. cyrtonema* Hua. by the nine-steaming and nine-basking approach. Then, 1,000 mg of crude 4-Z processed PCP was eluted through cellulose column chromatography using 0 M, 0.05 M, and 0.1 M NaCl solution to yield PCP-F0 (520 mg), PCP-F0.05 (110 mg), and PCP-F0.1 (270 mg), respectively, with a flow rate of 2.0 mL/min. The result was summarized in Supplementary Figure S1. According to DPPH radical assays, the PCP-F0.1 group at a concentration of 0.2, 0.6, and 1.0 mg/mL provided a scavenging rate of 3.7% ± 0.7, 7.1% ± 0.8, and 13.9% ± 0.9, respectively. These results were presented in Figure 3A. Additionally, according to the hydroxyl radical assays, the PCP-F0.1 group at a concentration of 0.2, 0.6, and 1.0 mg/mL provided a scavenging rate of 24.3% ± 0.2, 54.9% ± 0.8, and 80.2% ± 0.8, respectively. These results were presented in Figure 3B. Based on the results of radical assays, the PCP-F0.1 group exhibited higher radical-scavenging abilities than PCP-F0 and PCP-F0.05 groups.

#### 2.3.2. Total sugar, acid sugar, homogeneity, and FT-IR spectroscopy

**Figure 4:**
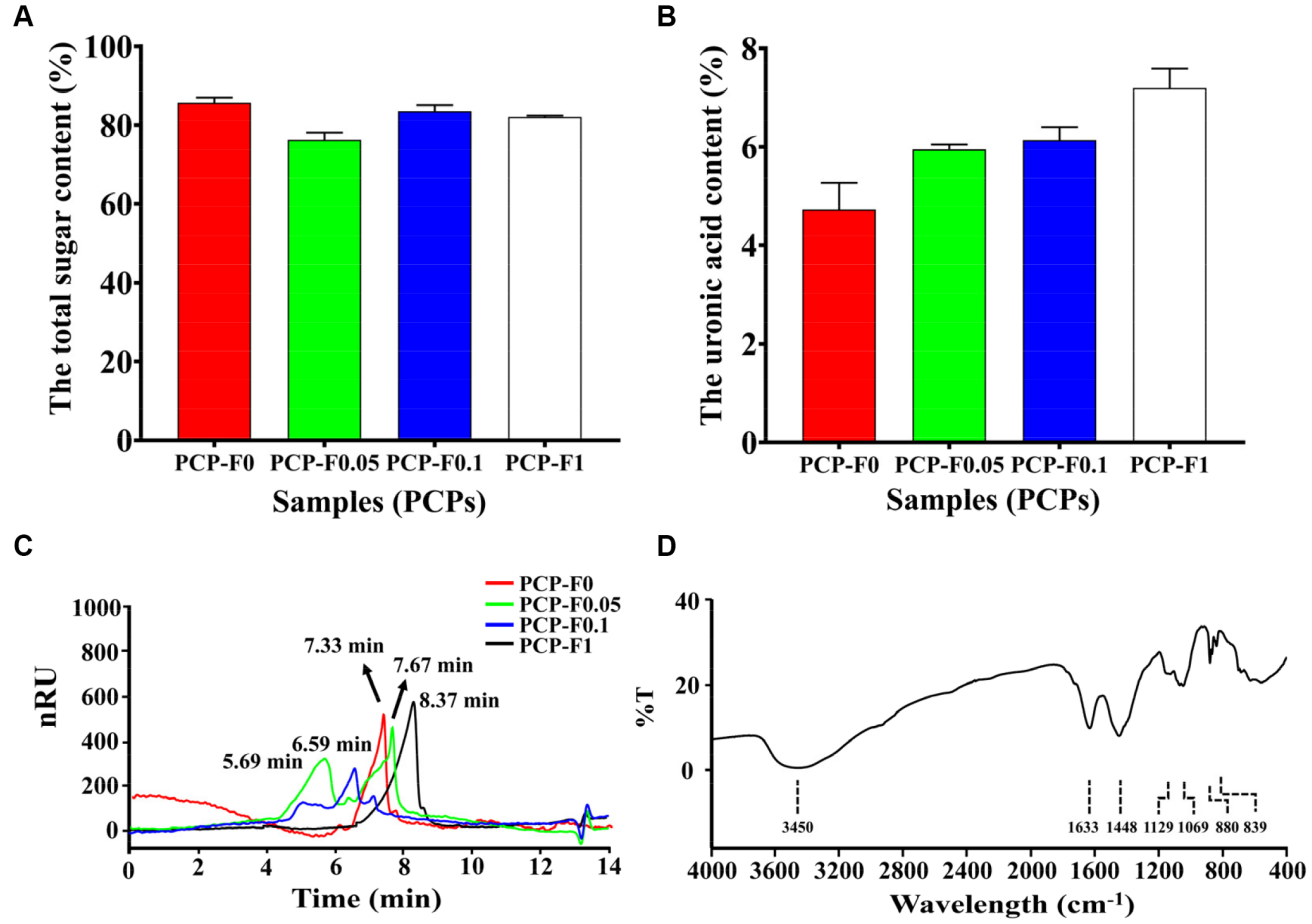
The characterization and physicochemical property analysis of samples (PCPs). The total sugar content **(A)**, the uronic acid content **(B)**, HPGPC **(C)**, and FTIR spectra of PCP-F1 **(D)**, (Tested three times at 25°C, *p* < 0.05).

The total sugar contents of PCP-F0, PCP-F0.05, PCP-F0.1, and PCP-F1 were 85.65 ± 1.30, 76.28 ± 1.77, 83.53 + 1.53, and 82.06 ± 0.35%, respectively. And the acid sugar contents of PCP-F0, PCP-F0.05, PCP-F0.1, and PCP-F1 were 4.73 ± 0.54, 5.95 ± 0.10, 6.14 ± 0.26, and 7.20 ± 0.39%, respectively (Figures 4A,B). There was no obvious absorption peak of polysaccharide PCP-F1 at 260 and 280 nm in Supplementary Figure S3, indicating that the amount of protein and nucleic acid impurity has been reduced to the lowest level, which is less than the minimum detection limit (35). The molecular weights of PCP-F0, PCP-F0.05, PCP-F0.1, and PCP-F1 were estimated as 107.51, 567.86 + 76.24, 228.53, and 37.46 kDa, respectively, based on the high-performance gel permeation chromatography (HPGPC) analysis (Figure 4C).

To understand the types of functional groups contained in molecule PCP-F1 and how they are connected, infrared spectroscopy is used. As shown in Figure 4D, the stretching vibration of O-H residues appears at 3,403, 1,626, and 1,452 cm^−1^, which are the bending vibration peaks of-COOH (27). The absorption peaks at 1,131 cm^−1^ represent the asymmetric stretching vibration of C-O-C (36). The absorption peak at 1,026 cm^−1^ belongs to the C-O-H bond. In addition, the absorption peaks at 880 and 835 cm^−1^ belong to β-configuration pyranose (30).

#### 2.3.3. Monosaccharide composition and methylation analysis

**Figure 5:**
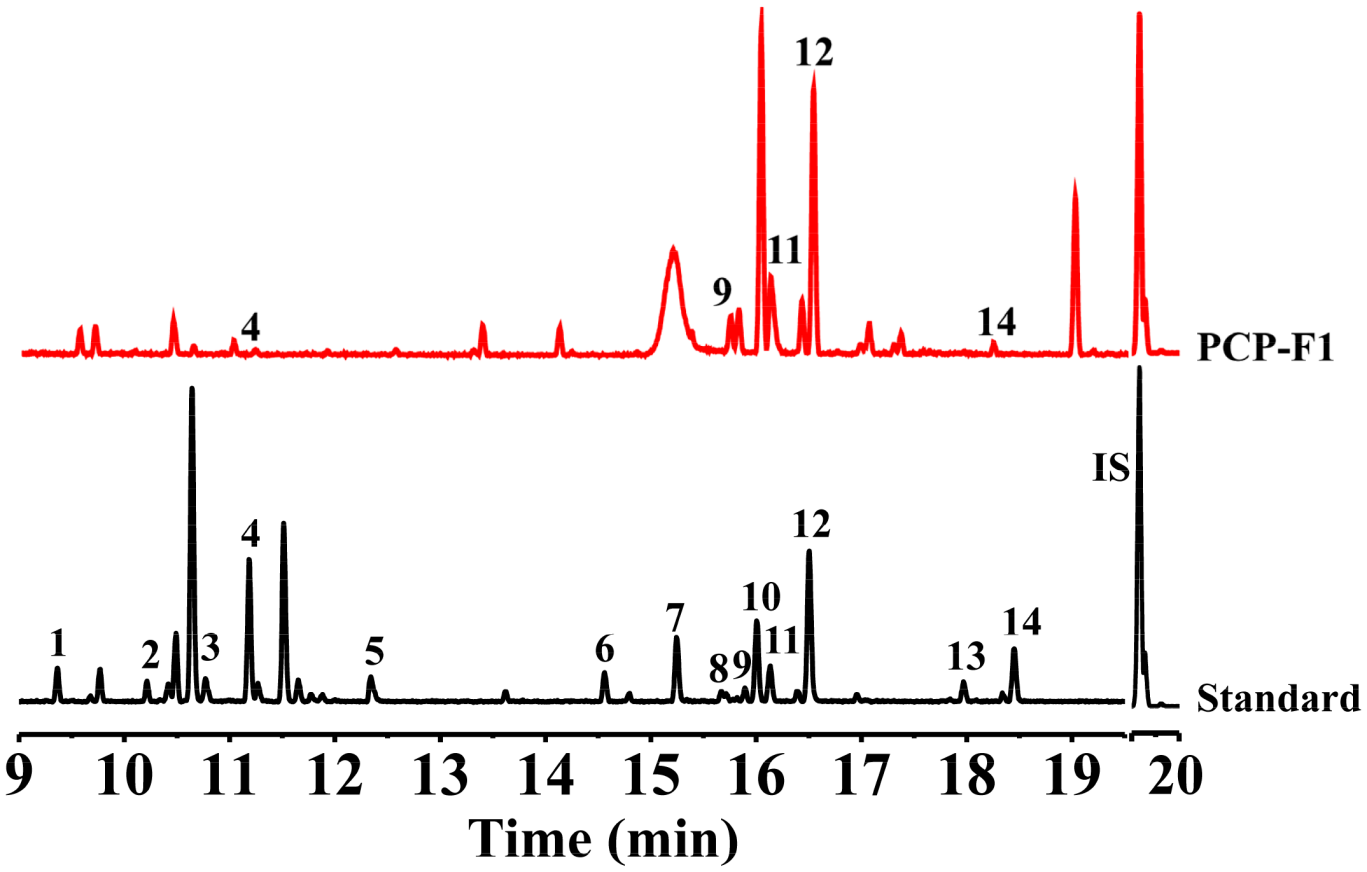
Monosaccharide composition analysis of PCP-F1. Peak 1, Xyl (T*_R_* = 9.36 min); Peak 2 and Peak 4, *ꞵ*-L-Rha (T*_R_* = 10.47 min) and *α*-L-Rha (T*_R_* = 11.17 min), respectively; Peak 3, Ara (T*_R_* = 10.60 min); Peak 5, Rib (T*_R_* = 12.30 min); Peak 6 and Peak 9, *ꞵ*-D-Glc (T*_R_* = 14.54 min), and *α*-D-Glc (T*_R_* = 15.90 min), respectively; Peak 7, Fru (T*_R_* = 15.20 min); Peak 8 and Peak 11, *ꞵ*-D-Man (T*_R_* = 16.12 min) and *α*-D-Man (T*_R_* = 15.70 min), respectively; Peak 10 and Peak 12, *ꞵ*-D-Gal (T*_R_* = 16.00 min) and *α*-D-Gal (T*_R_* = 16.50 min), respectively; Peak 13, GlcA (T*_R_* = 17.92 min); Peak 14, GalA (T*_R_* = 18.40 min); IS: T*_R_* = 19.68 min.

The monosaccharide composition of PCP-F1 was Glc, Man, Gal, Rha, and GalA with a molar ratio of 3.5: 2.5: 1.3: 1.8: 0.8 (Figure 5). By methylation and GC–MS analysis of PCP-F1, the methylation results of glycoside linkage PCP-F1 were determined, as shown in Table 1 and Supplementary Figure S4. The type of glycoside linkage PCP-F1 was determined by the MS fragment database and literature analysis (37). Methylation analysis revealed that PCP-F1 was composed of →3) Glc*p*, →1) Man*p* (3→, →3) Rha*p* (4→, →2) Glc*p* (3→, →1) Glc*p* (2→, →2) Glcp (6→, →2) Gal*p* (4→, →2, 4) Man*p* (6→, →2) GalA*p* (3, 4→/→2) Gal*p* (3, 4 → with a molar percentage of 2.4: 13.3: 13.4: 6.9: 24.2: 1.8: 18.5: 11.8: 7.7.

**Table 1:** Methylation analysis of PCP-F1.

No.	Methylation product	T_R_ (min)	Linkage type	Main MS (m/z)	Molar ratio (mol%)
1	3,5-di-O-acetyl-2,4,6-tri-O-methyl glucitol	6.7	→3)Glc*p*	74,101,116,88,59	2.4
2	1,3,5-tri-O-acetyl-2,4,6-tri-O-methyl mannitol	6.9	→1)Man*p*(3→	71,87,102,129,59	13.3
3	3,4,5-tri-O-acetyl-2,6-O-methyl rhamnitol	7.2	→3)Rha*p*(4→	74,116,85,101	13.4
4	2,3,5-tri-O-acetyl-4,6-di-O-methyl glucitol	12.4	→2)Glc*p*(3→	101,71,128,143,59	6.9
5	1,2,5-tri-O-acetyl-1,3,6-tri-O-methyl glucitol	12.8	→1)Glc*p*(2→	87,71,144,59,101	24.2
6	2,5,6-tri-O-acetyl-3,4-di-O-methyl glucitol	14.5	→2)Glc*p*(6→	73,115,128,88,58	1.8
7	2,4,5-tri-O-acetyl-3,6-di-O-methyl galactitol	17.2	→2)Gal*p*(4→	115,73,58,84,98	18.5
8	2,3,5,6-tetra-O-acetyl-4-O-methyl mannitol	18.9	→2,4)Man*p*(6→	87,129,74,112,99	11.8
9	2,3,4,5-tetra-O-acetyl-1-O-methyl galactitol	20.1	→2)Gal*p*(3,4→/→2)GalA*p*(3,4→	103,71,60,90,85	7.7

#### 2.3.4. Partial acid hydrolysis

Due to the complexity of polysaccharide structure, the partial acid hydrolysis of PCP-F1 was performed further to determine the distribution of monosaccharides. In polysaccharide skeletons, the hydrolysis of the branch chain is a priority than that of the backbone (27). In this study, PCP-F1 was divided into four subcomponents (H-1, H-2, H-3, and H-4), and the monosaccharide components of these subcomponents are shown in Table 2. Due to the Man, Gal, and Glc being the main monosaccharide composition of H-1, they are presumed to locate in the side chain. When the concentration of TFA increases to 0.1 M, the contents of Glc and Gal in H-2 are higher than others. Meanwhile, GalA was determined in H-2. In the structurally stable H-4, the Glc, Man, and Gal were found, indicating that they should be located in the core structure of PCP-F1.

**Table 2:** Monosaccharide composition of the fractions after partial acid hydrolysis.

Monosaccharides	Monosaccharide composition (%, m/m)			
	H-1	H-2	H-3	H-4
Glc	47.8	48.6	43.6	70.4
Rha	1.3	/	3.0	/
Man	23.7	7.8	24.0	14.0
Gal	27.3	36.6	22.1	15.5
GalA	/	6.9	7.3	/

#### 2.3.5. NMR analysis

Nuclear Magnetic Resonance (NMR) spectroscopy is a widely used and important technique for ascertaining distinct structural attributes. It is employed for verifying monosaccharide classifications, distinguishing between α-and β-anomeric arrangements, and unraveling details about glycosidic connections (38–40). Herein, the ^13^C and ^1^H NMR spectra of residues A to I were analyzed through 2D NMR. Regarding the information gleaned from the ^13^C and ^1^H spectra of PCP-F1, the specific signals corresponding to different residues are outlined as follows.

Within the 1H NMR spectrum depicted in Supplementary Figure S5A, the presence of a signal at δ_H_ 4.62 ppm suggests the presence of a *β*-configured residue within the PCP-F1 structure. This signal is attributed to H-1 of residue D (38). Moreover, the regions between δ_H_ 4.05 and δ_H_ 3.43 ppm were responsible for H-2 to H-6 of residues (A, B, C, D, E, F, G, H, and I) presented in PCP-F1 (39). In ^13^C NMR spectra depicted in Supplementary Figure S5B, nine peaks at δ_C_ 106.47, 92.68, 104.33, 104.28, 102.29, 96.37, 106.06, 103.78, and 104.35 ppm were assigned as the anomeric signals situated at C-1 of residues A–I, respectively. In the more elevated field segment, the resonance at δ_C_ 166.73 ppm was attributed to the carbonyl group of the uronic acid moiety (22).

**Figure 6:**
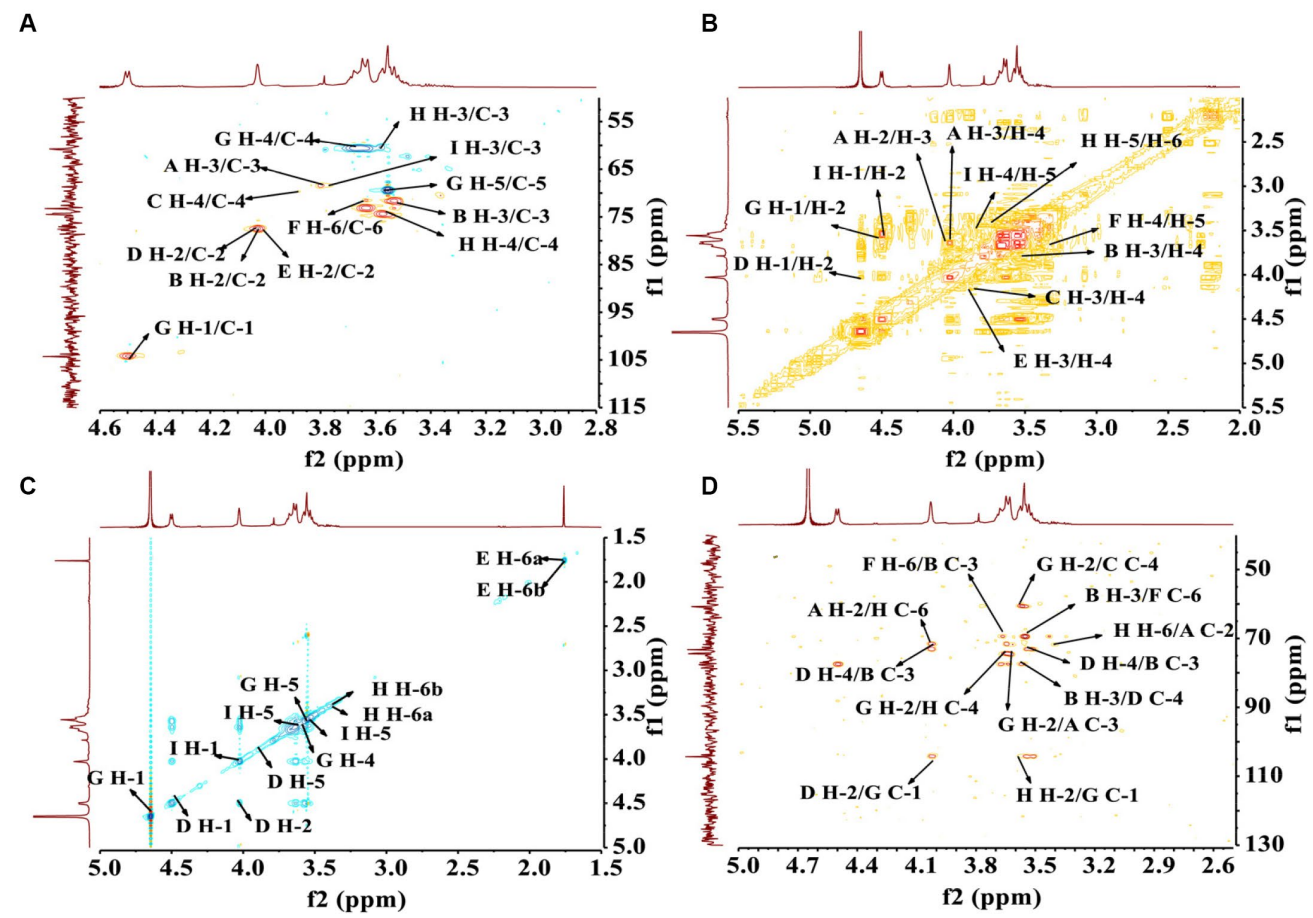
The structural elucidation of PCP-F1 by 2D NMR analysis. **(A)** HSQC spectrum; **(B)** ^1^H-^1^H COSY spectrum; **(C)** NOESY spectrum; and **(D)** HMBC spectrum.

In order to establish the correlation between the signals of the anomeric carbon and their respective protons, an analysis of the HSQC spectrum depicted in Figure 6A was conducted. First, the signals at δ_H/C_ 4.31/104.28 and 4.51/106.06 ppm were attributable to heterologous region H-1/C-1 of the residue D and G, respectively (40). The ^1^H-^1^H COSY (Figure 6B) and NOESY (Figure 6C) spectrum revealed that these cross-peaks at δ_H_ 4.62/4.02, 4.02/3.81, 3.81/3.96, 3.96/3.79, and 3.79/3.68 ppm were consistent with the H-2 to H-6 signal of residue D at δ_H_ 4.02, 3.81, 3.96, 3.79, and 3.68 ppm, respectively. In addition, six cross peaks at δ_H_ 4.65/3.60, 3.60/3.73, 3.73/3.68, 3.68/3.55, and 3.55/3.53 ppm which were consistent with the H-2 to H-6 signal of residue G at δ_H_ 3.60, 3.73, 3.68, 3.55, and 3.53 ppm, respectively.

The HMBC spectrum facilitated the analysis of correlations among nine residues (A, B, C, D, E, F, G, H, and I). As depicted in Figure 6D, a reciprocal relationship was observed between the H-2 signal of residue A (δ_H_ 4.00 ppm) and the C-6 of residue H (δ_C_ 77.91 ppm), as well as between the H-6 signal of residue H (δ_H_ 3.43 ppm) and the C-2 of residue A (δ_C_ 71.76 ppm), signifying a presence of a 2,6-linkage between residue A and H. Similarly, a positive interaction was noted between the C-3 signal of residue B (δ_C_ 74.42 ppm) and the H-6 of residue F (δ_C_ 3.61 ppm), and between the C-6 signal of residue F (δ_C_ 71.53 ppm) and the H-3 of residue B (δ_H_ 3.56 ppm), indicating a 3,6-linkage between residue B and F.

Further examinations revealed the existence of 1,2-linkages between residue G and both F and H. The C-1 signal of residue G (δ_C_ 106.06 ppm) displayed a positive correlation with the H-2 of residue F (δ_H_ 3.96 ppm), and the C-2 signal of residue F (δ_C_ 74.92 ppm) exhibited a correlation with the H-1 of residue G (δ_H_ 4.65 ppm), illustrating the 1,2-linkages between residue G and F. Additionally, the C-1 signal of residue G (δ_C_ 106.06 ppm) showed a positive correlation with the H-2 of residue H (δ_H_ 3.62 ppm), and the C-2 signal of residue H (δ_C_ 73.09 ppm) correlated with the H-1 of residue G (δ_H_ 4.65 ppm), indicating 1,2-linkages between residue G and H. The linkage type between residue B and D was determined to be a 3,4-linkage based on the highly correlated H-3 signal of residue B (δ_H_ 3.56 ppm) and the C-4 of residue D (δ_C_ 73.17 ppm), as well as the correlation between the C-3 signal of residue B (δ_C_ 74.42 ppm) and the H-4 of residue D (δ_H_ 3.96 ppm). Similarly, the linkage type between residue G and D was identified as a 1,2-linkage, with the H-1 signal of residue G (δ_H_ 4.65 ppm) correlating with the C-2 of residue D (δ_C_ 77.41 ppm), and the C-1 signal of residue G (δ_C_ 106.06 ppm) showing correlation with the H-2 of residue D (δ_H_ 4.02 ppm).

Lastly, a 2,3-linkage between residue E and I was established, as evidenced by the correlation between the C-2 signal of residue E (δ_C_ 77.39 ppm) and the H-3 of residue I (δ_H_ 3.82 ppm), along with the correlation between the H-2 signal of residue E (δ_H_ 3.98 ppm) and the C-3 of residue I (δ_C_ 88.05 ppm). A comprehensive assignment of PCP-F1 was outlined in Table 3.

**Table 3:** Signal assignment in the ^1^H and ^13^C NMR spectra of PCP-F1.

	Glycosyl residues	H-1/C-1	H-2/C-2	H-3/C-3	H-4/C-4	H-5/C-5	H-6/C-6
A	→2)-α-D-GalAp(3,4→	−/106.47	4.00/71.76	3.84/74.46	3.73/77.51	3.96/−	166.73
B	→3) -α-D-Glcp	−/92.68	4.03/77.41	3.56/74.42	3.67/61.79	3.80/68.44	3.51/66.44
C	→3) -α-L-Rhap(4→	−/104.33	3.49/62.45	3.83/−	3.99/74.92	4.01/−	1.76/23.07
D	→2) -ꞵ-D-Galp(4→	4.62/104.28	4.02/77.41	3.81/68.44	3.96/73.17	3.79/60.96	3.68/61.79
E	→2) -α-D-Glcp(3→	−/102.29	3.98/77.39	3.83/−	4.05/−	3.48/62.45	3.50/−
F	→2) -α-D-Glcp(6→	−/96.37	3.96/74.92	3.81/68.44	3.44/65.11	3.73/−	3.61/71.53
G	→1) -ꞵ-D-Glcp(2→	4.65/106.06	3.60/77.51	3.73/−	3.68/71.59	3.55/69.43	3.53/60.62
H	→2,4)-α-D-Manp(6→	−/103.78	3.62/73.09	3.59/60.29	3.58/74.42	3.63/71.59	3.43/77.91
I	→1) -ꞵ-D-Manp(3→	4.45/104.35	3.48/62.45	3.82/88.05	3.85/−	3.64/71.43	3.63/73.09

#### 2.3.6. Congo red experiment

**Figure 7:**
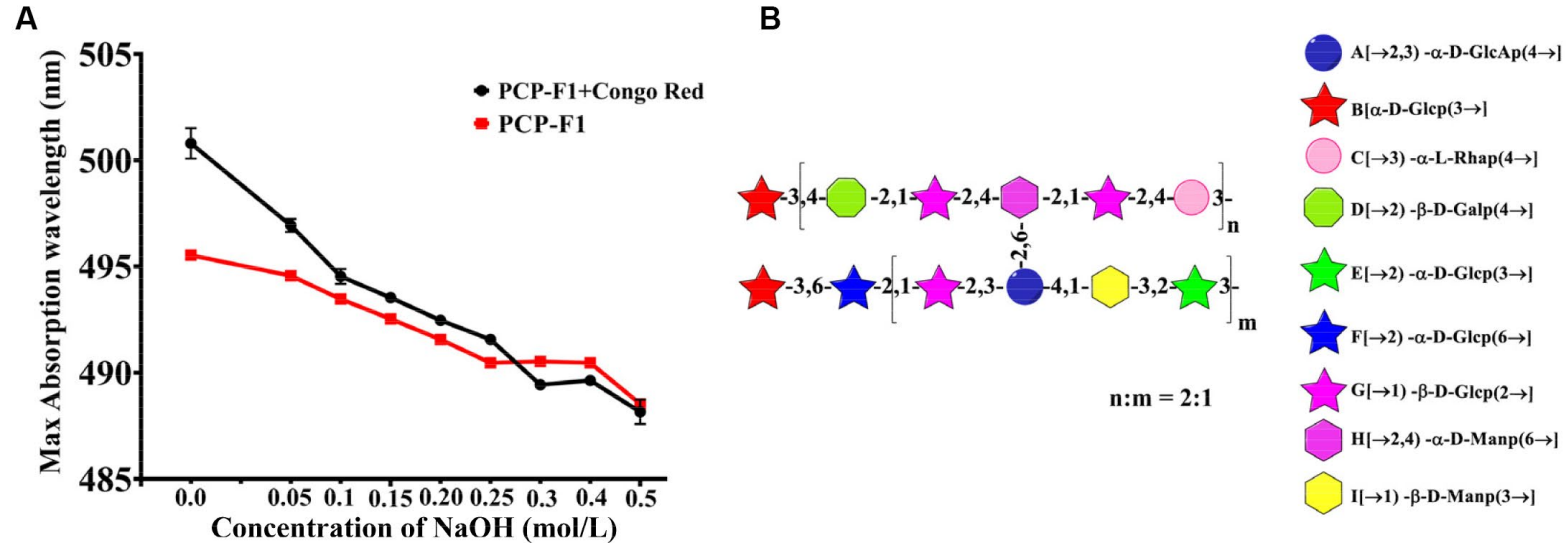
The structural analysis of PCP-F1. The Congo red **(A)** and putative structure **(B)**. (Tested three times at 25°C, *p* < 0.05).

The triple helix structure of PCP-F1 was characterized by Congo red experiment. Under weakly alkaline conditions, the triple helical polysaccharide can form a complex with Congo red, and compared with Congo red solution, the maximum absorption wavelength will be red-shifted (41). With the increase of concentration of alkali, the complex triple helix structure will be destroyed, leading to the decrease of the maximum absorption wavelength. As shown in Figure 7A, the maximum absorption wavelength of PCP-F1 was 496 nm. Compared with the Congo red solution, the maximum absorption wavelength of PCP-F1 polysaccharide showed a weak red-shift trend, indicating that no triple helix conformation was formed.

#### 2.3.7. Structural deduction

Monosaccharides and glycoside chains are structural components associated with polysaccharide activity. These structural characteristics include composition, conformation, molecular weight, and functional groups. Combined with these results and previous reports (38–42), the structure of PCP-F1 was tentatively determined to be a heteroglycan comprised of nine individual residues with side chains →3)-*α*-D-Glc*p*, →2)-*α*-D-Glc*p* (6→, →1)-*ꞵ*-D-Glc*p* (2→, →2)-*α*-D-GalA*p* (3,4→, →1) -*ꞵ*-D-Manp (3→, →2)-*α*-D-Glc*p* (3→, branched located at O-3 position of →3)-*α*-D-Glc*p*, →2*)-ꞵ-*D-Gal*p* (4→, →1)-*ꞵ*-D-Glc*p* (2→, →2,4)-*α*-D-Man*p* (6→, →3)-*α*-L-Rha*p* (4→, and corresponding structural motif is depicted in Figure 7B.

## 3. Discussion

### 3.1. Physicochemical properties of processed PCPs

The “nine-steaming and nine-basking” approach is one of the traditional methods of processing precious and nourishing Chinese medicine. Subjecting the herb to multiple rounds of steaming and drying, typically around nine cycles, imparts a gently “warm” quality to it. This transformation renders the herb apt for alleviating symptoms associated with “cold” conditions. In most cases, raw rhizomes are not employed in their untreated state. Instead, the practice of Nine-Steam Nine-Bask is favored, as it effectively augments the herb’s tonic attributes via a series of sequential steaming and drying processes. Through the analysis of processed PCPs, including total sugar, acid sugar and FT-IR spectroscopy, and pH measurement, and combined with the result of antioxidant activity, 4-Z PCPs displayed a better antioxidant performance in all the processed PCPs group and thus were selected for the next experiment.

### 3.2. Structure characteristics of PCP-F1

Under the action of stomach acid, large molecular-weight polysaccharides are easily degraded into small molecular-weight polysaccharides, oligosaccharides, and even monosaccharides. For monosaccharide composition, galactose has a positive effect on hydroxyl radical scavenging activity, while galacturonic acid and rhamnose have a negative effect. Mannose, glucuronic acid, and glucose have no significant effect on antioxidant activity (43). In addition, β-structured polysaccharides in the form of pyranose have higher antioxidant activity (44). The PCP-F1 contains a large amount of Glc, Man, Gal, and a small amount of Rha, GalA, and small contents of Glc, Man, and Gal were β configuration.

Previous studies reported the change and dynamic analysis of monosaccharide composition and content of processed PCPs (10–12, 45–47), but there was no further structural analysis. First, we used the procedure of steaming and basking nine times, combined with the water extraction and alcohol precipitation method to obtain the crude processed PCPs. By radical scavenging assay, the 4-Z PCPs showed the best activity. The DEAE-52 cellulose and Sephadex G-200 column were used to yield PCP-F1. Finally, UV, HPGPC, IR, GC–MS, and NMR were used to analyze the structure of PCP-F1. The UV showed that PCP-F1 had no nucleic acid or protein. Monosaccharide composition analysis showed that PCP-F1 is mainly composed of Glc, Man, Gal, Rha, and GlcA, consistent with the results of methylation analysis and partial acid hydrolysis. Combined with the above results, the structure of PCP-F1 was suggested: →3)-*α*-D-Glc*p*, →2)-*α*-D-Glc*p* (6→, →1)-*ꞵ*-D-Glc*p* (2→, →2)-*α*-D-GalA*p* (3,4→, →1) -*ꞵ*-D-Manp (3→, →2)-*α*-D-Glc*p* (3→, branched located at O-3 position of →3)-*α*-D-Glc*p*, →2*)-ꞵ-*D-Gal*p* (4→, →1)-*ꞵ*-D-Glc*p* (2→, →2,4)-*α*-D-Man*p* (6→, →3)-*α*-L-Rha*p* (4 → .

### 3.3. The structure and antioxidant activity relationship

The relationship between the structure of polysaccharides and their antioxidant activity has been studied and analyzed. DPPH radical scavenging is in terms of the hydrogen supply ability of antioxidants, and polysaccharides play an antioxidant role by providing H+ to free radicals to stop the oxidation reaction (48). The content of uronic acid can be an important indicator of the antioxidant activity of polysaccharides, that is, polysaccharides with high uronic acid content often have strong antioxidant activity. Moreover, polysaccharides with high content in GalA can exhibit significant antioxidant activity, possibly due to their-COOH functional groups (49). The increase of galactose content in polysaccharides may enhance their antioxidant activity. The number of active hydroxyl groups in polysaccharide structure positively correlates with its ability to scavenge radicals (50). In addition, molecular weight is another significantly vital factor affecting antioxidant activity, and polysaccharides with molecular weights ranging from 4,000 to 100,000 Da exhibit high DPPH scavenging activity. Low-molecular-weight polysaccharides may have more reducing hydroxyl groups at their ends, which can accept and scavenge free radicals (51).

We performed the DPPH and hydroxyl radical assay to study *in vitro* antioxidant activity of PCP-F1. The EC50 of DPPH scavenging rate of PCP-F0, PCP-F0.05, PCP-F0.1, and PCP-F1 were 6.54, 4.83, 4.1, 0.96, and 0.48 mg/mL, respectively. And the EC50 of hydroxyl radicals scavenging rate of PCP-F0, PCP-F0.05, PCP-F0.1, and PCP-F1 were 5.88, 0.58, 0.53, and 0.48 mg/mL, respectively. The results showed that PCP-F1 could scavenge radicals with a higher scavenging rate. The contents of uronic acid of PCP-F0, PCP-F0.05, PCP-F0.1, and PCP-F1 were 4.73 ± 0.54, 5.95 ± 0.10, 6.14 ± 0.26, and 7.20 ± 0.39% respectively, corresponding to the antioxidant activity of PCP-F0 < PCP-F0.05 < PCP-F0.1 < PCP-F1. The lowest molecular weight of PCP-F1 was 37.46 kDa between 4,000 and 100,000 Da and PCP-F1 contained 18.5% → 2)-*ꞵ*-D-Galp (4 → and 7.7% → 2)-*α*-D-GalAp (3,4→. Therefore, the stronger antioxidant activity of PCP-F1 is the result of multiple factors such as its high content of uronic acid and low molecular weight. We will further investigate its antioxidant mechanism and reveal its potential structure–activity relationship.

## 4. Materials and methods

### 4.1. Materials and chemicals

The rhizome of *P. cyrtonema* Hua. was obtained from Jinzhai County, China, and authenticated by Prof. Ruonan Xie, Anhui University of Chinese Medicine. Sigma Chemical Company provided dextrans of varying molecular weights for the experiment. A variety of sugars, including L-rhamnose (Rha), D-glucose (Glc), D-fructose (Fru), L-arabinose (Ara), D-galactose (Gal), D-xylose (Xyl), D-mannose (Man), and D-galacturonic acid (GalA) were commercially available purchased. The Millipore system provided distilled water. Kuer Co., Ltd. provided Sephadex G-200 and DEAE cellulose-52. Anthrone, 1,1-diphenyl-2-picrylhydrazyl (DPPH), hydrogen peroxide, Trifluoroacetic acid (TFA), 3-phenyl phenol, Sodium tetraborate, and Dimethyl sulfoxide were provided by Shanghai Macklin Biochemical Co., Ltd. N-Hexadecane-D34 (C16H34) was purchased from Aladdin industrial corporation. Anhydrous ethanol was bought from Jiangsu Qiangsheng Functional Chemistry Co., Ltd. Starter 3100 pH meter was obtained from Aarhus Instruments (Changzhou) Co., Ltd. SHIMADZU spectrophotometer was bought from Shimadzu International Trade (Shanghai) Co., Ltd. The GC–MS was obtained from Brook Dalton Company.

### 4.2. Preparation and purification of PCPs

Nine-steaming and nine-basking approach: according to previously reported (22), *P. cyrtonema* was washed and placed in a steamer to steam for 12 h. The sample was dried overnight in the dryer (setting at 50°C) and collected as the sample 1-Z. Then half of the sample 1-Z was taken to steam under the above conditions to prepare 2-Z. Similarly, 3-Z, 4-Z, 5-Z, 6-Z, 7-Z, 8-Z, and 9-Z were collected, respectively. The raw *P. cyrtonema* was marked as 0-Z. Processed PCPs were isolated from steamed *P. cyrtonema* (0-Z, 1-Z, 2-Z, 3-Z, 4-Z, 5-Z, 6-Z, 7-Z, 8-Z, and 9-Z) using water decocting (1, 4 w/v, 100°C) for 1 h. Moreover, ethanol precipitation (the final concentration of ethanol was 80% with four times volumes) was used to remove small molecular impurities such as oligosaccharides. The precipitates were then deproteinized according to Sevag’s method. The purified PCPs were collected and named 0-Z PCPs, 1-Z PCPs, 2-Z PCPs, 3-Z PCPs, 4-Z PCPs, 5-Z PCPs, 6-Z PCPs, 7-Z PCPs, 8-Z PCPs, and 9-Z PCPs for further analysis.

Purification of the 4-Z PCPs was performed on a DEAE-52 Cellulose column (3.5 cm × 30 cm), with gradient NaCl solutions (0, 0.05, 0.1, 0.2, 0.3, 0.4, and 0.5 M). *In vitro* antioxidant activities were used to screen the active fraction, and the elution fractions, PCP-F1 (from 0.1 M NaCl) was then separated using deionized water on a Sephadex G-200 column (1.6 cm × 40 cm), and was gathered for further analysis.

### 4.3. Characterization and structural analysis

#### 4.3.1. pH experiments and FT-IR spectrum of processed PCPs

Processed PCPs solution with a concentration of 1 mg/mL was prepared and tested at 25°C by Starter 3100 pH meter. Processed PCPs and PCP-F1 were, respectively, mixed with dried potassium bromide (1, 100, w/w) to ground and pressed in a vacuum at 25°C. A Nicolet 5700 IR spectrometer given corresponding spectra in the wavelength region of 4,000–400 cm^−1^.

#### 4.3.2. Total sugar and uronic acid determination of processed PCPs

The uronic acid content and total sugar content of processed PCPs were measured by the anthrone-sulfuric acid and m-hydroxyphenyl methods, respectively (27, 30).

#### 4.3.3. UV–visible spectrum, monosaccharide composition, and homogeneity of PCP-F1

PCP-F1 solution (1 mg/mL) was prepared and scanned by a SHIMADZU spectrophotometer at a wavelength range of 200–800 nm at 25°C. Monosaccharide composition analysis was conducted employing Gas Chromatography-Single Quadrupole Mass Spectrometry (GC–MS) methodology. Briefly, PCP-F1 (20 mg) was introduced into a solution of trifluoroacetic acid (TFA) with a concentration of 2 mol/L and a volume of 2 mL. Subsequently, hydrolysis was performed at 100°C for a duration of 6 h, followed by evaporation of the solvent under reduced pressure. The acid-hydrolyzed analyte, dissolved in anhydrous DMSO (2 mL), was combined stepwise with NaOH powder (60 mg) in a flask. Following stirring at 35°C for 30 min, methylating reagent CH_3_I (1 mL) was carefully added drop by drop. The reaction proceeded in darkness for 12 h, after which it was halted by introducing ultrapure water (2 mL). The resulting methylated sample was subjected to extraction using dichloromethane and subsequently subjected to analysis via GC–MS. PCP-F1 homogeneity was determined based on a previous method (22).

#### 4.3.4. Methylation analysis of PCP-F1

Glycosidic linkage analysis of PCP-F1 was conducted with a slight modification to a previously documented method (35, 37, 52). The initial step involved reducing the uronic acid to a neutral sugar prior to methylation analysis. To achieve this, 10 mg of dried PCP-F1 was fully dissolved in anhydrous DMSO (2 mL). Subsequently, NaOH (60 mg) was added, and the reaction mixture was stirred at 35°C for a span of 2 h. Following this, a slow addition of 1 mL of CH_3_I took place. The reaction proceeded under dark conditions for 12 h. Termination of the reaction occurred by adding ultrapure water (2 mL). The resultant methylated sample underwent extraction using dichloromethane and was washed thrice with ultrapure water. Complete methylation of PCP-F1 was confirmed by the disappearance of peaks in the infrared spectrum within the range of 3,200–3,700 cm^−1^. The ensuing procedure involved hydrolyzing the mixture using 2 mL of 2 M TFA at 100°C for a period of 6 h. Post removal of excess TFA, NaBH_4_ (30 mg) and a solution of 0.05 M NaOH (1 mL) were sequentially introduced. After a reaction time of 12 h, 100 μL of acetic acid was added, followed by solvent removal under vacuum. Subsequently, a mixture of pyridine and acetic anhydride (1 mL each) was added, sealed, and stirred at 90°C for an additional 2 h. The reacted solution was subjected to extraction using dichloromethane and subsequently analyzed using the GC–MS technique.

#### 4.3.5. Partial acid hydrolysis of PCP-F1

According to a previous method (27), partial acid hydrolysis of PCP-F1 was performed. 20 mg PCP-F1 was hydrolyzed with 2 mL TFA for 1 h (0.05 M, 100°C). TFA was removed, and four volumes of ethanol (95%) were added to the hydrolysate. The sample was stored at 4°C overnight for centrifugation (5,000 rpm, 5 min), the supernatant was collected and named H-1, and the precipitation was hydrolyzed again with 2 mL TFA (0.1 M, 100°C) for 1 h. Similarly, after removing the TFA with methanol, the supernatant was obtained and named H-2. The precipitation was separated and hydrolyzed with 2 mL TFA with a concentration of 0.5 mol/L at 100°C for 1 h to get H-3 and the final precipitated H-4. The H-1, H-2, H-3, and H-4 samples were completely hydrolyzed using 2 mL TFA (2 M, 100°C) for 6 h to further analysis.

#### 4.3.6. NMR spectra of PCP-F1

Nuclear magnetic resonance (NMR) measurements were performed using 0.6 mL of D_2_O dissolved with 20 mg of dry polysaccharide PCP-F1 at 25°C. A 600 Hz NMR spectrometer was utilized to record the 1D ^1^H NMR and ^13^C NMR, as well as the 2D ^1^H-^1^H COSY, HSQC, HMBC, and NOESY spectra of the PCP-F1.

#### 4.3.7. Congo red experiment of PCP-F1

According to a previous method (41), the Congo red experiment of PCP-F1 was performed. The polysaccharide solution (1 mL, 1 mg/mL) and Congo Red solution (100 μM) at the ratio of 1: 1 (v/v) were mixed with different concentrations of NaOH (1 mL, 0, 0.05, 0.10, 0.15, 0.20, 0.25, 0.30, 0.40, and 0.50 M) at 25°C for 30 min, and the final solution was determined at a wavelength of 400–600 nm.

### 4.4. Antioxidant assay

#### 4.4.1. DPPH free radical scavenging

The capacity of scavenging DPPH radicals of processed PCPs and purified 4-Z PCPs, including PCP-F0, PCP-F0.05, PCP-F0.1, and PCP-F1, were detected according to a previous report with slight modifications (36). Briefly, first, fresh DPPH (0.2 mM in methanol, 2.0 mL) and PCPs solutions (2.0 mL) at various concentrations (0, 0.2, 0.4, 0.6, 0.8, and 1.0 mg/mL) were prepared. Second, the reaction mixture of prepared DPPH and PCPs solutions at various concentrations was thoroughly stirred and hatched at 25°C for 30 min under dark conditions. The UV-2550 UV spectrophotometer recorded the absorbance of the mixture at a wavelength of 517 nm. Finally, the activity of DPPH radical scavenging was calculated.

#### 4.4.2. Hydroxyl radical scavenging

The capacity of scavenging hydroxyl radicals of processed PCPs and purified 4-Z PCPs, including PCP-F0, PCP-F0.05, PCP-F0.1, and PCP-F1, were measured refer to a previous report (53). First, PCPs solutions (1.0 mL) at various concentrations (0.2, 0.4, 0.6, 0.8, 1.0, 2.0, 4.0, 6.0, 8.0, and 10.0 mg/mL) were prepared. Second, the reaction mixture of prepared sample solutions (1 mL), FeSO_4_ solution (1 mL, 6 mM), H_2_O_2_ solution (1 mL, 6 mM), and salicylic acid (1 mL, 2 mM in ethanol) was mixed thoroughly and incubated at 37°C for 30 min. The UV-2550 ultraviolet spectrophotometer determined the absorbance of the mixture to a wavelength of 510 nm. Finally, the activity of hydroxyl radical scavenging was measured.

## 5. Conclusion

A highly potent and uniform polysaccharide, PCP-F1, exhibiting superior antioxidant capabilities, was successfully derived from the 4-Z PCPs. The structural characteristics of PCP-F1 were meticulously elucidated via a comprehensive approach involving 2D NMR investigations coupled with partial acid hydrolysis. The fundamental backbone of PCP-F1 was composed of →3)-*α*-D-Glc*p*, →2)-*α*-D-Glc*p* (6→, →1)-*ꞵ*-D-Glc*p* (2→, →2)-*α*-D-GalA*p* (3,4→, →1) -*ꞵ*-D-Man*p* (3→, →2)-*α*-D-Glc*p* (3→, while being adorned with side chains including →3)-*α*-D-Glc*p*, →2)-*ꞵ*-D-Gal*p* (4→, →1)-*ꞵ*-D-Glc*p* (2→, →2,4)-*α*-D-Man*p* (6→, →3)-*α*-L-Rha*p* (4 → branches situated at the O-3 position of →2)-*α*-D-GalA*p* (3,4→. The assessment of antioxidant prowess exhibited by PCP-F1 unveiled its remarkable ability to neutralize DPPH and hydroxyl radicals within the concentration range of 0–1.0 M. This finding underscores the promising potential of PCP-F1 as a valuable constituent in the realm of functional foods.

## Data availability statement

The original contributions presented in the study are included in the article/Supplementary material, further inquiries can be directed to the corresponding authors.

## Author contributions

YZ: Investigation, Writing – original draft, Formal Analysis, Methodology. ZW: Investigation, Writing – original draft, Formal Analysis. RF: Formal Analysis, Writing – original draft. RX: Project administration, Writing – review & editing. BW: Conceptualization, Project administration, Funding acquisition, Supervision, Writing – review & editing. QL: Funding acquisition, Writing – review & editing.

## Funding

The author(s) declare financial support was received for the research, authorship, and/or publication of this article. We are grateful to the Natural Science Foundation of Anhui Province (2008085MH271) and the Talent support project of Anhui University of Chinese Medicine (2019rczd002). This work was supported by the Anhui Collaborative Innovation Project (GXXT-2020-025) and Overseas Study Project for Outstanding Young Talents (gxgwfx2020039).

## Conflict of interest

The authors declare that the research was conducted in the absence of any commercial or financial relationships that could be construed as a potential conflict of interest.

## Publisher’s note

All claims expressed in this article are solely those of the authors and do not necessarily represent those of their affiliated organizations, or those of the publisher, the editors and the reviewers. Any product that may be evaluated in this article, or claim that may be made by its manufacturer, is not guaranteed or endorsed by the publisher.

## References

[1] Liu J Si J. Herbal textual research on Chinese medicine "Huangjing" (Polygonati Rhizoma) and some enlightenments. Zhongguo Zhong Yao Za Zhi. (2018) 43:631–6. doi: 10.19540/j.cnki.cjcmm.20180105.001, PMID: 29600633

[2] Hu Y Yin M Bai Y Chu S Zhang L Yang M. An evaluation of traits, nutritional, and medicinal component quality of Polygonatum cyrtonema Hua and *P. sibiricum*. Red Front Plant Sci. (2022) 13:891775. doi: 10.3389/fpls.2022.891775, PMID: 35519815 PMC9062581

[3] Zhao P Zhou H Zhao C Li X Wang Y Wang Y. Purification, characterization and immunomodulatory activity of fructans from Polygonatum odoratum and P. cyrtonema. Carbohydr Polym. (2019) 214:44–52. doi: 10.1016/j.carbpol.2019.03.014, PMID: 30926006

[4] Li L Thakur K Cao Y Liao B Zhang J Wei Z. Anticancerous potential of polysaccharides sequentially extracted from Polygonatum cyrtonema Hua in human cervical cancer Hela cells. Int J Biol Macromol. (2020) 148:843–50. doi: 10.1016/j.ijbiomac.2020.01.223, PMID: 31982521

[5] Li J Wang X Zhou R Cheng F Tang X Lao J. Polygonatum cyrtonema Hua polysaccharides protect BV2 microglia relief oxidative stress and Ferroptosis by regulating NRF2/HO-1 pathway. Molecules. (2022) 27:7088. doi: 10.3390/molecules27207088, PMID: 36296678 PMC9610736

[6] Li X Ma R Zhang F Ni Z Thakur K Wang S. Evolutionary research trend of Polygonatum species: a comprehensive account of their transformation from traditional medicines to functional foods. Crit Rev Food Sci Nutr. (2021) 63:3803–20. doi: 10.1080/10408398.2021.1993783, PMID: 34669530

[7] Ren H Deng Y Zheng J Ye X Xia L Liu M. Integrated phytochemical analysis based on UPLC-Q-TOF-MS/MS, network pharmacology, and experiment verification to explore the potential mechanism of *Platycodon grandiflorum* for chronic bronchitis. Front Pharmacol. (2020) 11:4163–82. doi: 10.3389/fphar.2020.564131, PMID: 33013400 PMC7506058

[8] Liu J Zhang Z Huai X Wei Y Zhu J Li X. Development and application of the new integrated equipment and process of the nine-steam-nine-bask method in the processing of Polygonatum cyrtonema. PRO. (2022) 10:1044. doi: 10.3390/pr10061044

[9] Luo L Qiu Y Gong L Wang W Wen R. A review of Polygonatum mill. Genus: its taxonomy, chemical constituents, and pharmacological effect due to processing changes. Molecules. (2022) 27:4821. doi: 10.3390/molecules27154821 35956772 PMC9369890

[10] Wu W Huang N Huang J Wang L Wu L Wang Q. Effects of the steaming process on the structural properties and immunological activities of polysaccharides from Polygonatum cyrtonema. J Funct Foods. (2022) 88:104866. doi: 10.1016/j.jff.2021.104866

[11] Jin J Lao J Zhou R He W Qin Y Zhong C. Simultaneous identification and dynamic analysis of saccharides during steam processing of rhizomes of Polygonatum cyrtonema by HPLC-QTOF-MS/MS. Molecules. (2018) 23:2855. doi: 10.3390/molecules23112855, PMID: 30400172 PMC6278431

[12] Chen Z Zhu B Chen Z Cao W Wang J Li S. Effects of steam on polysaccharides from Polygonatum cyrtonema based on saccharide mapping analysis and pharmacological activity assays. Chin Med. (2022) 17:97. doi: 10.1186/s13020-022-00650-3, PMID: 35978410 PMC9386940

[13] Vaccaro A Kaplan Dor K Nambara K Pollina E Lin C Greenberg M. Sleep loss can cause death through accumulation of reactive oxygen species in the gut. Cells. (2020) 181:1307–1328.e15. doi: 10.1016/j.cell.2020.04.049, PMID: 32502393

[14] Shadel G Horvath T. Mitochondrial ROS signaling in organismal homeostasis. Cells. (2015) 163:560–9. doi: 10.1016/j.cell.2015.10.001, PMID: 26496603 PMC4634671

[15] Hou Y Xie Z Cui H Lu Y Zheng T Sang S. Trapping of glyoxal by propyl, octyl and dodecyl gallates and their mono-glyoxal adducts. Food Chem. (2018) 269:396–403. doi: 10.1016/j.foodchem.2018.07.030, PMID: 30100451

[16] Wang L Li L Gao J Huang J Yang Y Xu Y. Characterization, antioxidant and immunomodulatory effects of selenized polysaccharides from dandelion roots. Carbohydr Polym. (2021) 260:117796. doi: 10.1016/j.carbpol.2021.117796, PMID: 33712144

[17] Ji X Guo J Cao T Zhang T Liu Y Yan Y. Review on mechanisms and structure-activity relationship of hypoglycemic effects of polysaccharides from natural resources. Food Sci Human Wellness. (2023) 12:1969–80. doi: 10.1016/j.fshw.2023.03.017

[18] Wang B Liu X Zhou A Meng M Li Q. Simultaneous analysis of coumarin derivatives in extracts of Radix Angelicae pubescentis (Duhuo) by HPLC-DAD-ESI-MSn technique. Anal Methods. (2014) 6:7996–8002. doi: 10.1039/C4AY01468E

[19] Wang Z Liu H Cao Y Zhang T Guo H Wang B. A novel method for investigating the mechanism of the anti-rheumatoid arthritis activity of Angelicae pubescentis radix by integrating UHPLC–QTOF/MS and network pharmacology. Biomed Chromatogr. (2022) 36:e5389. doi: 10.1002/bmc.5389, PMID: 35484722

[20] Wang L Wang Y Tong G Li Y Lei M Wu H. Development of a novel UHPLC-UV combined with UHPLC-QTOF/MS fingerprint method for the comprehensive evaluation of Nao-Luo-Xin-Tong: multi-wavelength setting based on traditional Chinese medicinal prescription composition. Anal Methods. (2019) 11:6092–102. doi: 10.1039/C9AY01975H

[21] Jiang H Liu J Wang Y Chen L Liu H Wang Z. Screening the Q-markers of TCMs from RA rat plasma using UHPLC-QTOF/MS technique for the comprehensive evaluation of Wu-Wei-Wen-Tong capsule. J Mass Spectrom. (2021) 56:e4711. doi: 10.1002/jms.4711, PMID: 33764633

[22] Wang Z Xie R Wang B. Comprehensive evaluation and anti-inflammatory activity of “Zhi” polygonatum cyrtonema produced by the classical steaming approach. Pharmacolog Res Mod Chin Med. (2023) 6:100229. doi: 10.1016/j.prmcm.2023.100229

[23] Shen F Song Z Xie P Li L Wang B Peng D. Polygonatum sibiricum polysaccharide prevents depression-like behaviors by reducing oxidative stress, inflammation, and cellular and synaptic damage. J Ethnopharmacol. (2021) 275:114164. doi: 10.1016/j.jep.2021.114164, PMID: 33932516

[24] Wang Z Sun Q Zhao Y Du J Wang B. Synthesis of naphthalimide-type chemsensor and its application in quality evaluation for polygonatum sibiricum red. Front Chem. (2022) 10:969014. doi: 10.3389/fchem.2022.969014, PMID: 36034663 PMC9402912

[25] Liu H Zhao Y Chen L Du J Guo H Wang B. A novel method for the pre-column derivatization of saccharides from Polygonatum cyrtonema Hua. By integrating Lambert–beer law and response surface methodology. Molecules. (2023) 28:2186. doi: 10.3390/molecules28052186, PMID: 36903433 PMC10004654

[26] Kumar S Lahlali R Liu X Karunakaran C. Infrared spectroscopy combined with imaging: a new developing analytical tool in health and plant science. Appl Spectrosc Rev. (2016) 51:466–83. doi: 10.1080/05704928.2016.1157808

[27] Shi Y Ye Y Zhang B Liu Y Wang J. Purification, structural characterization and immunostimulatory activity of polysaccharides from Umbilicaria esculenta. Int J Biol Macromol. (2021) 181:743–51. doi: 10.1016/j.ijbiomac.2021.03.176, PMID: 33798575

[28] Ji X Cheng Y Tian J Zhang S Jing Y Shi M. Structural characterization of polysaccharide from jujube (*Ziziphus jujuba* mill.) fruit. Chem Biol Tech Agric. (2021) 8:54. doi: 10.1186/s40538-021-00255-2

[29] Su Y Li L. Structural characterization and antioxidant activity of polysaccharide from four auriculariales. Carbohydr Polym. (2020) 229:115407. doi: 10.1016/j.carbpol.2019.115407, PMID: 31826485

[30] Ji X Hou C Yan Y Shi M Liu Y. Comparison of structural characterization and antioxidant activity of polysaccharides from jujube (*Ziziphus jujuba* mill.) fruit. Int J Biol Macromol. (2020) 149:1008–18. doi: 10.1016/j.ijbiomac.2020.02.018, PMID: 32032709

[31] Anraku M Gebicki J Iohara D Tomida H Uekama K Maruyama T. Antioxidant activities of chitosans and its derivatives in vitro and in vivo studies. Carbohydr Polym. (2018) 199:141–9. doi: 10.1016/j.carbpol.2018.07.016, PMID: 30143114

[32] Gebicki J. Oxidative stress, free radicals and protein peroxides. Arch Biochem Biophys. (2016) 595:33–9. doi: 10.1016/j.abb.2015.10.021 27095212

[33] Liang Z Pan Y Qiu L Wu X Xu X Shu Y. The changes of chemical constituents in the process of nine steaming and drying of Polygonatum polygonatum based on UPLC-Q-TOF-MS/MS. Chin Tradit Herbal Drugs. (2022) 53:4948–57. doi: 10.7501/j.issn.0253-2670.2022.16.004

[34] Gong G Fan J Sun Y Wu Y Liu Y Sun W. Isolation, structural characterization, and antioxidativity of polysaccharide LBLP5-a from *Lycium barbarum* leaves. Process Biochem. (2016) 51:314–24. doi: 10.1016/j.procbio.2015.11.013

[35] Zhang F Zhang L Chen J Du X Lu Z Wang X. Systematic evaluation of a series of pectic polysaccharides extracted from apple pomace by regulation of subcritical water conditions. Food Chem. (2021) 368:130833. doi: 10.1016/j.foodchem.2021.130833, PMID: 34425342

[36] Joshi K Kumar P Kataria R. Microbial carotenoid production and their potential applications as antioxidants: a current update. Process Biochem. (2023) 128:190–205. doi: 10.1016/j.procbio.2023.02.020

[37] Sims I Carnachan S Bell T Hinkley S. Methylation analysis of polysaccharides: technical advice. Carbohydr Polym. (2018) 188:1–7. doi: 10.1016/j.carbpol.2017.12.075, PMID: 29525144

[38] Wu Y Huo Y Xu L Xu Y Wang X Zhou T. Purification, characterization and antioxidant activity of polysaccharides from Porphyra haitanensis. Int J Biol Macromol. (2020) 165:2116–25. doi: 10.1016/j.ijbiomac.2020.10.053, PMID: 33069819

[39] Gao N Chen R Mou R Xiang J Zhou K Li Z. Purification, structural characterization and anticoagulant activities of four sulfated polysaccharides from sea cucumber *Holothuria fuscopunctata*. Int J Biol Macromol. (2020) 164:3421–8. doi: 10.1016/j.ijbiomac.2020.08.150, PMID: 32835799

[40] Guo Q Cui S Kang J Ding H Wang Q Wang C. Non-starch polysaccharides from American ginseng: physicochemical investigation and structural characterization. Food Hydrocoll. (2015) 44:320–7. doi: 10.1016/j.foodhyd.2014.09.031

[41] Wang Y Li X Chen X Zhao P Qu Z Ma D. Effect of stir-frying time during Angelica Sinensis Radix processing with wine on physicochemical, structure properties and bioactivities of polysaccharides. Process Biochem. (2019) 81:188–96. doi: 10.1016/j.procbio.2019.02.020

[42] Yang J Shen M Wu T Chen X Wen H Xie J. Physicochemical, structural characterization, and antioxidant activities of chondroitin sulfate from *Oreochromis niloticus* bones. Food Sci. (2023) 12:1102–8. doi: 10.1016/j.fshw.2022.10.027

[43] Yi Y Lamikanra O Sun J Wang L Min T Wang H. Activity diversity structure-activity relationship of polysaccharides from lotus root varieties. Carbohydr Polym. (2018) 190:67–76. doi: 10.1016/j.carbpol.2017.11.090, PMID: 29628261

[44] Zhang L Hu Y Duan X Tang T Shen Y Hu B. Characterization and antioxidant activities of polysaccharides from thirteen boletus mushrooms. Int J Biol Macromol. (2018) 113:1–7. doi: 10.1016/j.ijbiomac.2018.02.084, PMID: 29458100

[45] Fan B Wei G Gan X Li T Qu Z Xu S. Study on the varied content of Polygonatum cyrtonema polysaccharides in the processing of steaming and shining for nine times based on HPLC-MS/MS and chemometrics. Microchem J. (2020) 159:105352. doi: 10.1016/j.microc.2020.105352

[46] Zheng X Xu C Jin C Liu J Liu C Li L. Study on the correlation between the cooking temperature and internal and external quality of "nine steaming and nine drying" Polygonatum polygonatum essence based on color change. Chin Tradit Herbal Drugs. (2022) 53:1719–29. doi: 10.7501/j.issn.0253-2670.2022.06.014

[47] Wang W Zhang X Dabu X He J Yang S Chen J. Analysis of chemical constituents from Polygonatum cyrtonema after “nine-steam-nine-bask” processing. Phytochem Lett. (2019) 29:35–40. doi: 10.1016/j.phytol.2018.11.004

[48] Zhong R Wan X Wang D Zhao C Liu D Gao L. Polysaccharides from marine Enteromorpha: structure and function. Trends Food Sci Technol. (2020) 99:11–20. doi: 10.1016/j.tifs.2020.02.030

[49] Li Z Nie K Wang Z Luo D. Quantitative structure activity relationship models for the antioxidant activity of polysaccharides. PLoS One. (2016) 11:e0163536. doi: 10.1016/j.tifs.2020.02.030, PMID: 27685320 PMC5042491

[50] Duan G Yu X. Isolation, purification, characterization, and antioxidant activity of low-molecular-weight polysaccharides from Sparassis latifolia. Int J Biol Macromol. (2019) 137:1112–20. doi: 10.1016/j.ijbiomac.2019.06.177, PMID: 31271800

[51] Liu X Liu H Yan Y Fan L Yang J Wang X. Structural characterization and antioxidant activity of polysaccharides extracted from jujube using subcritical water. Lwt. (2020) 117:108645. doi: 10.1016/j.lwt.2019.108645

[52] Ji X Guo J Ding D Gao J Hao L Guo X. Structural characterization and antioxidant activity of a novel high-molecular-weight polysaccharide from *Ziziphus Jujuba* cv. J Food Measure Charact. (2022) 16:2191–200. doi: 10.1007/s11694-022-01288-3

[53] Zhao Z Huangfu L Dong L Liu S. Functional groups and antioxidant activities of polysaccharides from five categories of tea. Ind Crop Prod. (2014) 58:31–5. doi: 10.1016/j.indcrop.2014.04.004

