# Supplementary material for: Structural characterization and antioxidant activity of processed polysaccharides PCP-F1 from Polygonatum cyrtonema Hua
Source: Front Nutr. 2023 Sep 5;10:1272977. doi: 10.3389/fnut.2023.1272977 (PMC10508638; doi:10.3389/fnut.2023.1272977)
Supplement: Supplementary file 1 [file Data_Sheet_1.docx]

***Abstract:*** Background: *Polygonatum cyrtonema* Hua. (PC) is a traditional Chinese herb with a history of use in both food and medicine. For clinical use, processed PC pieces are most commonly used, while present research has focused on crude PC polysaccharides (PCPs). Methods: In this study, a new polysaccharide, PCP-F1, with a molecular weight of 37.46 kDa, was separated from four-time processed PCPs by column chromatography and evaluated by antioxidant activity. It was composed of glucose, mannose, galactose, rhamnose and galacturonic acid with a molar ratio of 3.5:2.5:1.3:1.8:0.8. Results and Discussion: The methylation analysis and two-dimensional NMR measurement revealed that the configuration of PCP-F1 contained nine residues in the primary structural unit by the chain of →3)-α-D-Glcp, →2)-α-D-Glcp(6→, →1)-ꞵ-D-Glcp(2→, →2)-α-D-GalAp(3,4→, →1) -ꞵ-D-Manp(3→, →2)-α-D-Glcp(3→, branched for →3)-α-D-Glcp, →2)-ꞵ-D-Galp(4→, →1)-ꞵ-D-Glcp(2→, →2,4)-α-D-Manp(6→, →3)-α-L-Rhap(4→. Radical scavenging assays indicated that PCP-F1 could scavenge radicals with a high scavenging rate, suggesting PCP-F1 possess good antioxidant activity. The study confirms the importance of processed PC and offers the potential for exploiting it as a functional food.
